# Menstrual cycles and the impact upon performance in elite British track and field athletes: a longitudinal study

**DOI:** 10.3389/fspor.2024.1296189

**Published:** 2024-02-20

**Authors:** Benjamin P. Jones, Ariadne L'Heveder, Charlotte Bishop, Lorraine Kasaven, Srdjan Saso, Sarah Davies, Robin Chakraverty, James Brown, Noel Pollock

**Affiliations:** ^1^ Department of Gynaecology, Hammersmith Hospital, Imperial College NHS Trust, London, United Kingdom; ^2^ Department of Metabolism, Digestion and Reproduction, Imperial College London, London, United Kingdom; ^3^ Lister Fertility Clinic, The Lister Hospital, HCA Healthcare, London, United Kingdom; ^4^ Department of Cutrale and Perioperative Ageing Group, Imperial College London, London, United Kingdom; ^5^ Women's Health, West Middlesex University Hospital, Chelsea and Westminster NHS Foundation Trust, Isleworth, United Kingdom; ^6^ National Performance Institute, British Athletics, Loughborough, United Kingdom; ^7^ Institute of Sport, Exercise and Health, University College London, London, United Kingdom

**Keywords:** gynaecology, exercise, athletes, menstrual cycles, menstrual disorders, amenorrhea

## Abstract

**Objective:**

To assess the prevalence of menstrual disorders and the perceived effect of menstrual cycles upon performance in elite athletes.

**Methodology:**

A longitudinal survey in the form of a questionnaire was sent to female track and field athletes at British Athletics every 6 months, over a five-year period between 1st October 2014 and 1st October 2019 in the United Kingdom (UK).

**Results:**

128 athletes completed an average of 4.2 ± 2.9 questionnaires across the study period. The mean age of menarche was 14.2 ± 1.4 years, 13.4 ± 1.3 years and 12.8 ± 1.4 years in endurance, power, and thrower athletes respectively (*p* < 0.05). Two-thirds (66%; *n* = 82) reported consistently regular cycles, 30% (*n* = 37) irregular at some point during the period of observation and 4% (*n* = 5) were amenorrhoeic. 87 athletes (68%) reported dysmenorrhoea and 40 (31%) menorrhagia. More than three quarters (76.8%; *n* = 96) described their cycle negatively affected performance. Amongst those who reported when the negative impact occurred (*n* = 91), 40% (*n *= 36) reported this in the late luteal phase and 35% (*n* = 32) during the early follicular phase. 79% (*n* = 100) of athletes reported at least one cyclical symptom, of which bloating, lower back and pelvic pain were most frequently experienced.

**Conclusion:**

This data highlights the complex interrelationship between women's health and elite athletic performance. Athletes perceive a negative impact from their menstrual cycles upon performance with a desire to manage these more effectively, particularly during competition. Female reproductive health expertise in the multi-disciplinary management of elite athletes is required.

## Introduction

Over the last sixty years, the advancement of sex equality has greatly enhanced both educational and professional opportunities for women. This subsequent empowerment of women has led to evolution in societal perceptions, including the breaking of barriers and gender stereotypes. Over this time, the participation of women in elite sports has increased significantly. During the Tokyo Olympics in 2020, for the first time ever, there was an equal number of men's and women's events, where 49% of athletes were female, compared to the Olympics in Rome in 1960, when just 11% of the athletes were female (1). Women's involvement in research has also been historically underrepresented, and as such there is a lack of knowledge about how female physiology changes throughout the menstrual cycle, and how this may impact performance. Whilst involvement has concurrently increased in recent years, data suggest that female participants in exercise-related research make up just over a third of participants (2). Concerningly, a recent study exploring the ratio of male and female participants in sport and exercise science research in the years 2014–2020, found 63% of publications included both males and females, 31% included only males, and a mere 6% included females only testing (3).

The physiological mechanisms controlling menstruation, with different levels of oestrogen and progesterone throughout the cycle, have previously been described by this group (4). Importantly, there is a growing body of evidence demonstrating menstrual cycle disorders in competitive female athletes (4, 5). Chronic low energy availability has been deemed to be one physiological mechanism explaining such disturbances, whereby energy imbalance leads to hypothalamic amenorrhea (4). This and a wider range of health conditions related to chronic low energy availability is referred to as Relative Energy Deficiency in Sport “REDs” (6). Indeed, amenorrhea has been shown to affect up to 65% of long-distance runners and 79% of ballet dancers respectively (7, 8), compared to 5% in the general population. High levels of physical activity have been associated with increased irregularity of periods, amenorrhea and a longer menstrual cycle (9–11). Concerning elite athletes, a study looking to characterize the menstrual status, body composition, and endocrine balance in female Olympic athletes, found that 27% of participants not using hormonal contraception experienced menstrual disturbances, mainly oligomenorrhea (12). A further observational prospective study found 40% of athletes experience menstrual cycle disorder (11). There is however limited evidence on menstrual cycle disturbance, and particularly the effect of menstruation on performance in British athletes, hence the need for this study.

Athletic performance is a multifaceted concept, comprising a variety of physical, psychological, emotional, and environmental variables (13). Whilst the primary effect of the menstrual cycle is on the reproductive system, the pulsatile release of ovarian hormones can impact physiological functioning throughout the body, and as such has the potential to positively or negatively impact sporting performance throughout the menstrual cycle (14). Whilst data remains scarce, it suggests that athletic performance is inconsistent across the cycle, and symptoms of menstruation itself can impact training and performance potential (15). As women compete at every phase of the menstrual cycle, and during menstruation itself, understanding its impact on athletes' performance, and whether there are any perceived or self-reported detrimental effects caused by the menstrual cycle is essential. The aim of this study is to assess for the prevalence of menstrual disorders and the perceived effect of menstrual cycles upon performance in elite British track and field athletes.

## Materials and methods

### Study design

Recruitment commenced from a population of elite track and field athletes at British Athletics over a five-year period between 1st October 2014 and 1st October 2019. Participants were sent a link, if they were on the elite world-class programme or were selected for an international Great Britain senior team, to an electronic questionnaire via email every six months throughout the study period. The questionnaire, (available in Supplementary Material), ascertained data on menstrual health including age at menarche, regularity of menstruation (self-defined by athletes as either regular or irregular), the number of days of bleeding they experienced and shortest and longest time between bleeding episodes. Further questions elicited information on how heavy and painful their menstrual periods were, the presence of cyclical symptoms and whether they experienced intermenstrual bleeding. Phases of the menstrual cycle were determined as follows: before bleeding; late luteal phase, during bleeding; early follicular phase, and earlier in the cycle; late follicular phase The final questions aimed to determine whether they perceived that their menstrual cycles negatively impacted their performance.

#### Ethics approval

Local institutional review board approval (6476/005) from University College London was obtained for the study and data was collected as part of usual clinical care of these patients by the British Athletics medical team.

#### Data analysis

Athletes were categorised into three groups depending on their discipline; power, endurance or throwing to allow subgroup analysis to be performed. Power athletes included those who competed in events such as sprinting (up to and including 400 m), jumping events and hurdles. Endurance athletes included athletes who ran in events from 800 m and above. The throwing group included those who competed in throwing field events. Reporting of results follows the CHAMP statement.

SPSS version 24 software [(IBM Corp, Armonk, NY, USA)] was used for analysis. Descriptive statistical analysis was described as mean ± SD. Menstrual dysfunction and age of menarche were compared between groups using Chi-squared test or Analysis of Variance as appropriate. Statistical significance was set at *p* < 0.05.

### Patient and public involvement

This study was developed as a direct response to the feedback from elite athletes regarding the impact of their menstrual cycle upon performance.

### Equity, diversity and inclusion

The author group is gender balanced and consists of junior, mid-career and senior researchers from gynaecological and sports medicine disciplines; however, all members of the author group are from one country. Our study population included female athletes only from different socioeconomic backgrounds participating in elite track and field athletics at the British Athletics Institute, Therefore, findings may not be generalizable to settings with fewer resources.

## Results

The questionnaire was sent to 208 athletes during the study period and 128 athletes responded at least once over the five-year period, resulting in a response rate of 61.5%. Each athlete responded a mean number of 4.2 ± 2.9 occasions throughout the study period. The mean age was 28 years ± 5.9. Two-thirds (66%; *n* = 85) of the athletes were white British, 21% (*n* = 27) were mixed and 13% (*n* = 16) were black British. Most athletes were power athletes (59%; *n* = 76), 27% (*n* = 35) were endurance athletes and 13% (*n* = 17) were throwers.

**Figure 1:**
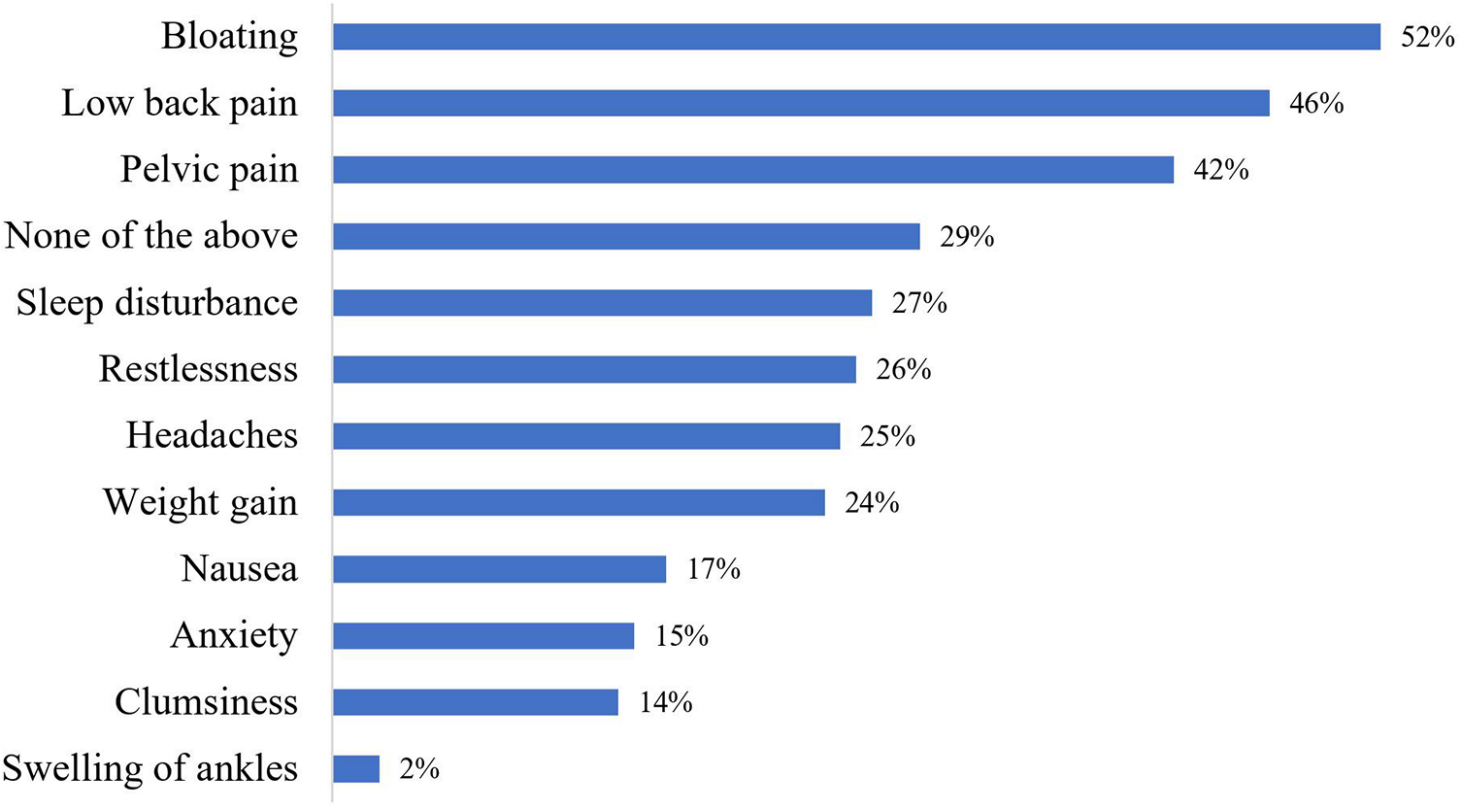
Proportion of athletes reporting specific cyclical symptoms.

The mean age at menarche for all athletes was 13.5 ± 1.4 years. The endurance athletes had a significantly later mean age of menarche at 14.2 ± 1.4 years, compared to power athletes who had a mean menarche of 13.4 ± 1.3 years, and the throwers who were the youngest at 12.8 ± 1.4 years (*p* < 0.05). Two-thirds of the athletes reported their cycles to be regular at every time of questioning (66%; *n* = 82), 30% (*n* = 37) reported their cycles to be irregular at some point throughout the period of observation and 4% (*n* = 5) reported themselves to be amenorrhoeic. The mean self-reported shortest gap in between menstruation was 25.2 ± 14.1 days (*n* = 104), and the mean longest gap was 51.7 ± 68.8 days (*n* = 110). In those who described their cycles as regular, 43% (*n* = 35) described inter-cycle variation of >5 days in the preceding six months. When subgroup analysis was performed, a greater proportion of endurance athletes experienced menstrual irregularities (irregular or absent menstruation), compared to the power and thrower subgroups (43% vs. 29% vs. 29% respectively), although the result was not statistically significant (*p* = 0.26). The mean duration of menses was 4.8 days (±1.6). 68% of athletes (*n* = 87) reported painful menstruation and 31% (*n* = 40) reported heavy menstrual bleeding. 79% (*n* = 100) of athletes reported that they experienced at least one cyclical symptom, a full list of symptoms in Supplementary Material and Figure 1. Regarding specific symptoms, as demonstrated in Figure 1, the most frequent included bloating, lower back pain and pelvic pain, which were reported by 52% (*n *= 66), 46% (*n* = 59) and 42% (*n* = 53) of athletes respectively.

**Figure 2:**
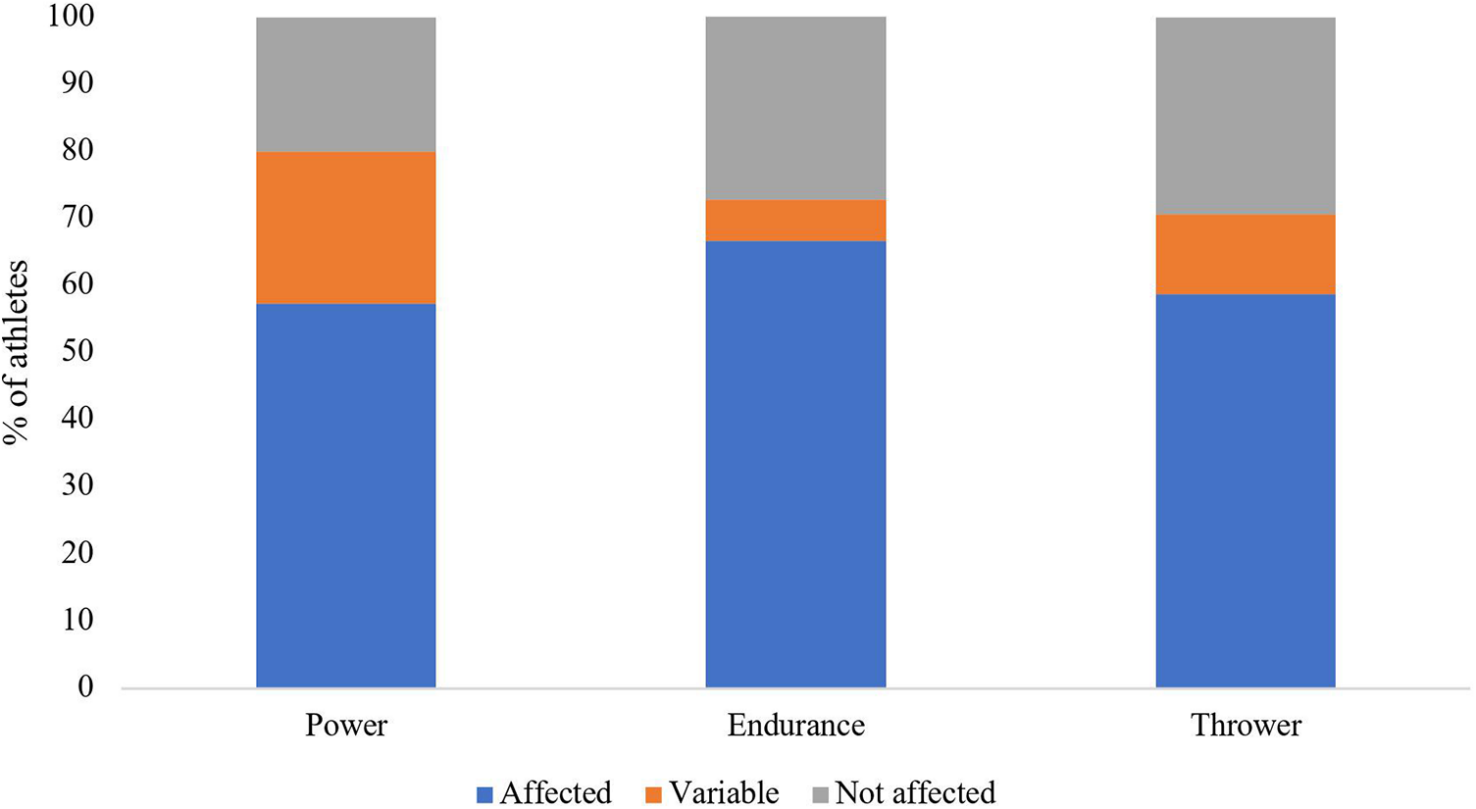
Effect of menstrual cycle on performance by event group.

125 athletes responded, at least once during the study period, to the question regarding whether their menstrual cycle affected their performance. More than three-quarters (77%; *n* = 96) reported their performance had been negatively impacted by their cycle at some point throughout the study period, whereas 60% (*n* = 75) of athletes reported that their performance was affected at every response. When compared amongst subgroups, as depicted in Figure 2, 71%–80% of athletes reported that their menstrual cycle affected performance during at least one time of questioning, with power athletes most affected amongst the three disciplines. 71% (*n* = 91) of athletes answered the question regarding what phase of the cycle they felt their performance was affected; 40% (*n* = 36) reported it as the time before menstruation (late luteal phase) and 35% (*n* = 32) reported it was during menstruation (early follicular phase), whereas 15% (*n* = 14) felt it was earlier in their cycle (late follicular phase) and 10% (*n* = 9) reported that it varied. When comparing those who had regular cycles (*n* = 80) with those who had irregular cycles (*n* = 41), 79% (*n* = 63) of those with regular cycles felt their performance had been affected by their cycles, compared to 76% (*n* = 31) of those with irregular cycles (*p* > 0.05).

**Figure 3:**
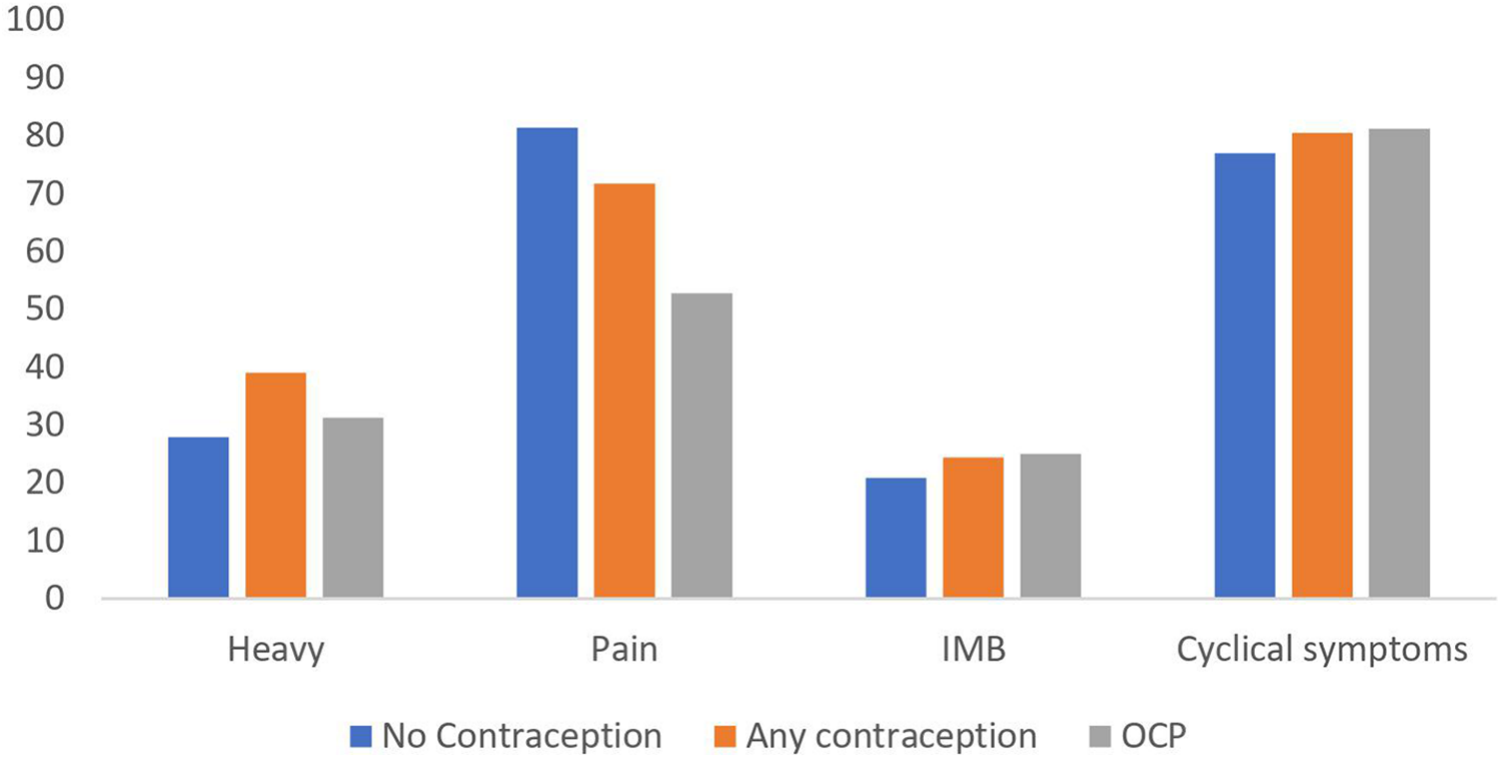
Proportion of athletes reporting symptoms of heavy menstrual bleeding, painful periods, irregular menstrual bleeding (IMB) and cyclical symptoms. (OCP, oral contraceptive pill).

Approximately one quarter (26.5%, *n* = 34) of athletes were taking contraception at every point of questioning, with a further 5.5% (*n* = 7) using it at some point during the study. 39% (*n* = 16) of these 41 athletes were using the combined oral contraceptive pill (COCP), four (10%) were using a progestogen intrauterine system (IUS), and the remaining 21 (51%) were using unspecified hormonal contraception. As summarised in Figure 3, there was no significant difference in heavy menstrual bleeding, painful menstruation, irregular bleeding or cyclical symptoms between women not taking contraception compared to those taking contraception, or specifically those taking the COCP (*p* > 0.05). With regards to whether their cycles impacted their performance, in those not taking any hormonal contraception, 78% (*n *= 64) felt their performance was negatively affected, compared to 80% (*n* = 32) in those taking hormonal contraception, and 87.5% (*n* = 14) in those specifically taking the COCP (*p* > 0.05).

Of the 83 athletes who responded to the question, 88% (*n* = 70) wanted additional input regarding the regulation of menstruation during competition. A further twelve (16%) wanted to discuss contraception, 10 (13%) wanted to discuss cyclical symptom management and 19 (24%) wanted to discuss other medical issues related to women's health.

## Discussion

This study aimed to assess the prevalence of menstrual disorders and the perceived effect of menstrual cycles upon performance in British elite athletes. It found that 30% of athletes (*n* = 37) reported an irregular cycle at some point during the period of observation and 4% (*n* = 5) were amenorrhoeic. A high proportion of athletes experienced troubling symptoms related to menstruation with 68% (*n* = 87) reporting dysmenorrhea and 31% (*n* = 40) reporting menorrhagia. Furthermore, 79% (*n* = 100) of athletes reported at least one cyclical symptom. Importantly, 76.8% (*n* = 96) described their cycle negatively affected performance.

In this study, more than a third of athletes reported irregular or absent menstruation. Even in those who described their cycles as regular, 43% (*n* = 35) demonstrated inter-cycle variation of >5 days in the preceding six months, suggesting high intra-variability of menstrual cycles. Variation in cycle length of >5 days has also been observed in studies in the general population, in as many as 52% of study participants, and thus this finding may not be exclusive to athletes (16). Athletes may however have a higher acceptance of menstrual irregularity and an unrealistic perception of what constitutes a regular cycle; indeed in a recent study amongst high school female athletes, 44% of the cohort believed that not having periods was a normal response to high training demands (17). The finding that those with menstrual irregularities demonstrated higher levels of anxiety, fatigue and pain inference than those without menstrual dysfunction, exemplifies the multi-faceted impact of menstrual cycle disruptions (17). It is therefore essential to address these issues, in order to optimise athlete health and performance.

The data presented herein also shows the mean age of menarche in this population was 13.5 years, which is later than that seen in a study of more than 80,000 women in the UK, where the mean age was 12.7 years (18). This is consistent with a systematic review which assessed physical activity and age at menarche, which found that menarche is delayed by 1.13 years in athletes, compared to their non-athletic counterparts (19). Moreover, we demonstrated herein that age at menarche is also influenced by discipline, with those athletes in the throwing subcategory having a mean age at menarche of 12.8, whereas endurance athletes had a significantly later age at menarche at 14.2 years. This is in line with a recent study which found the age of menarche in specifically endurance athletes was 14 years, compared to 12.5 in the non-athletic control group (20). This is of relevance given that delayed menarche can be a sign of REDs; the hypooestrogenic state may impair longterm bone health and have implications particularly from a sports injury perpesctive (4, 6).

### Clinical implications

In addition to gynaecological illness present in the general population, various mechanisms have been proposed to explain the increased rates of menstrual irregularity observed in athletes such as increased rates of polycystic ovaries (12). One of the most well researched explanations for menstrual disturbance is REDs (21). This is not a normal consequence of training load; but rather REDs describes a syndrome of interrelated health consequences which occurs when an individual's energy intake is insufficient to meet the demands of their body. It causes a range of physiological and psychological consequences, including decreased bone density, impaired immune function, menstrual dysfunction, decreased muscle strength and endurance. REDs supersedes the previously used term “female athlete triad”, which only considered the interrelationship between menstrual function, bone health and low energy availability (6, 22). Athletes are at risk of problematic low energy availability because they typically have high energy requirements due to the intense physical activity in which they engage, and they may not consume enough energy to compensate for their increased energy requirements. Some athletes may intentionally restrict their energy intake in an attempt to reduce body weight or body fat in sports that emphasise low body weight or aesthetics, such as gymnastics, figure skating, and endurance sports. Menstrual dysfunction in athletes predominantly clinically manifests in two main ways depending on the age at presentation; menstrual dysfunction in adults, or delayed menarche in pre-pubertal children (4). It is crucial that coaches and womens health specialists are alerted to athletes with delayed menarche and assess for and address any health concerns related to REDs in a multidisciplinary manner using the updated REDs Clinical Assessment Tool- Version 2 from the International Olympic Committee (6).

### Impact on performance

In our population, more than three-quarters of the cohort perceived that their menstrual cycle impacted their performance at some stage throughout the period of questioning, with 6 in 10 consistently reporting that it did at every visit. Three-quarters of the athletes reported that the part of their cycle where performance was negatively affected, was either before their menses, during the late luteal phase, or during their menses, in the early follicular phase. Despite the clear perception that menstrual cycles impact the performance of elite UK athletes demonstrated herein, a previous study reporting self-reported performance at different phases of the menstrual cycle amongst 241 elite athletes found no difference throughout the different phases (23). From a physiological perspective, oestrogen, which is the dominant sex hormone during the follicular phase, is thought to have beneficial effects on cardiovascular function by promoting vasodilation, whereas progesterone, the dominant hormone during the luteal phase, appears to have an antagonistic effect (15). Progesterone also has a thermogenic effect, with peak body temperature during the luteal phase (24), and a resulting negative impact on metabolic rate, causing thermoregulatory and cardiovascular strain (25). Oestrogen also promotes glucose uptake into muscles as a substrate for contraction, whereas progesterone inhibits this action (26). Finally, during exercise, oestrogen causes a preferential metabolism of free fatty acids over stored muscle glycogen, potentially preserving these stores during endurance events (15). As such, it would be logical to expect performance to be worse during periods when oestrogen levels are low, such as the late luteal or early follicular phases, which would appear to be in line with the data reported herein.

Despite these plausible physiological explanations and the findings from this study, there is no convincing clinical evidence that performance differs between phases of the menstrual cycle. Some studies found a significant increase in strength around the time of ovulation, which was attributed to the heightened oestrogen levels (27), but these results have not been replicated in other studies, such as those assessing maximum weight bench press weight and aerobic performance over 100–200 metre time trials (28). A recent meta-analysis investigating the effect of menstrual cycle phase on endurance and strength found a trivial reduction in performance during the early follicular phase compared to other phases, however due to low quality and quantities of research in this field, the results are largely inconclusive (29). Another systematic review assessed the impact of menstrual cycle phase on performance specifically in elite athletes, including 314 athletes across seven studies. The findings were inconsistent and insignificant, reaffirming that there is no clear evidence performance is affected by menstrual cycle phase (30). Clearly, there is a disparity between the self-reported perceived effect on performance throughout the menstrual cycle and actual physiological differences in performance. More high-quality data is required in this area to decipher the complex interaction between menstrual cycle phase and performance level.

To try and mitigate the potential physiological implications of fluctuating endogenous sex hormones, or at least the perception of inferior performance throughout the cycle, hormonal contraception may be used by athletes to control their cycles. Hormonal contraception can be used to delay the onset of menses until after a specific competition date, reduce cyclical symptoms or prevent iron deficiency anaemia in those with menorrhagia. By taking oral combined contraceptives in a tricyclic approach, some athletes may even be able to avoid having a withdrawal bleed during an entire competitive season. Despite the proposed benefits, there was no evidence of a perceived improvement in performance in those taking hormonal contraception in our cohort. This was confirmed by a recent meta-analysis which described a trivial, but likely negative impact of contraceptive use on athletic performance (31). The use of combined oral contraceptives has not been shown to improve muscle strength development when compared to non-users of hormonal contraceptives (32), and it has also been associated with a significant decrease in peak oxygen consumption (a marker for exercise capacity) (33). As such, whilst they may be required for family planning, or mitigating adverse symptoms of cyclical symptoms or menstruation, oral hormonal contraception should not be recommended routinely for the purpose of improving competitive performance. Our data identified that 81% of women not taking hormonal contraception reported painful menstruation, compared to 53% of those taking the COCP reporting the same symptom, although this was not statistically significant. Similarly, given the increase in women choosing to use a progestogen IUS as contraception, but also to reduce undesirable side effects such as dysmenorrhea, menorrhagia and irregular cycles, a similar pattern has emerged amongst athletes. Whilst there is good evidence progestogen IUS can improve menstrual pain in women with endometriosis (34), and improve menstrual bleeding in women with adenomyosis or fibroids (35), there is no data on the specific effects of the use of progestogen IUS on the mitigation of such symptoms in elite athletes. Moreover, there is limited data on the possible impact on athletic performance and side effects in such populations. As such, athletes should be presented with information regarding all contraceptive options, their administration, and their roles in managing a variety of gynaecological concerns which may be relevant to the individual, as well as their efficacy as a contraceptive agent to allow them to make an informed choice on contraception. There is no evidence that one particular agent should be universally recommended to elite athletes until further research has been undertaken.

Irrespective of the hormonal explanations for performance variation throughout the cycle, menses itself can cause anxiety, with the fear of flooding enough to distract them from competition and training (36). Given that 3 in 10 of the athletes presented herein describe heavy menstruation, this warrants consideration of management strategies to reduce menstrual flow or prevent menstruation in such women. Moreover, given that more than three-quarters of the athletes in this cohort reported cyclical symptoms, this reinforces the need to individualize management depending on the specific symptoms each athlete experiences. Finally, this study and others have shown menstrual dysfunction is clearly prevalent amongst athletes (9–12). It is crucial that these disturbances are not deemed acceptable and rather are investigated and managed by a multidisciplinary team throughout the athlete's career.

## Strengths and limitations

Whilst this appears to be largest prospective study assessing menstrual disorders and the effect of menstrual cycles upon performance in elite British track and field athletes, it has its limitations. Firstly, the questionnaire was not validated and therefore it is debatable whether the questions were optimal to elucidate the study questions. Notably, there is currently no validated questionnaire to assess menstrual cycles in athletes or their impact on performance. The questionnaire nature of the study results in subjectivity with certain responses, particularly regarding the impact of menstruation on performance. It also introduces recall bias. Regularity of menstruation was self-defined by athletes and although an attempt to obtain objective measurements of regularity was made by asking the longest and shortest duration between menstruation, this is subject to memory bias. As the study was undertaken on a finite number of elite British athletes, the small sample size amongst some subgroups may have resulted in reduced statistical power. Moreover, because the study focuses on elite athletes solely at British Athletics, this data is only extrapolatable to elite performers. Additionally, two thirds of the population were white British, as such, it is not a representation of the general population or other ethnicities. Future prospective, multi-centre international studies should be considered to increase the sample size and minimise potential bias.

## Conclusion

This study gives a unique insight into the menstrual cycles of elite British track and field athletes, by clearly highlighting the prevalence and subsequent impact of menstrual-related symptoms and irregularities. Despite the absence of high-quality data identifying any significant impact of menstrual phase upon performance, we clearly identify herein that top-level athletes perceive their performance to vary throughout the menstrual cycle, with the majority reporting inferior performance during the late luteal or early follicular phases, whilst circulating oestrogen and progesterone levels are low. The data presented underscores the need for more comprehensive support and education for female athletes regarding menstrual health, and the need for individualised, multi-disciplinary strategies to help mitigate the negative impact of menstrual dysfunction to optimise athlete health and performance.

## Data Availability

The raw data supporting the conclusions of this article will be made available by the authors, without undue reservation.
