# Supplementary material for: Menstrual cycles and the impact upon performance in elite British track and field athletes: a longitudinal study
Source: Front Sports Act Living. 2024 Feb 20;6:1296189. doi: 10.3389/fspor.2024.1296189 (PMC10912517; doi:10.3389/fspor.2024.1296189)
Supplement: Supplementary file 1 [file Datasheet1.docx]

**Menstrual cycles and the impact upon performance in elite UK athletes: a longitudinal study**

1. What event category do you perform in?

Power

Endurance

Throwing

1. What is your age?
2. What is your ethnicity?
3. How old were you when you had your first period?
4. Do you have regular cycles?

Yes

No

1. What is your average cycle length?
2. What is the longest Gap between your periods in the last 6 months?
3. What is the shortest gap in your periods in the last 6 months?
4. How many days on average do you bleed for?
5. Is your menstrual bleeding heavy?

Yes

No

1. Are your periods very painful?

Yes

No

1. Do you get bleeding between your periods?

Yes

No

1. Have you had a cervical smear test?

Yes

No

1. Is your performance affected during your menstrual cycle?

Yes

No

1. If so, when does this affect you?

Before your bleed

During your bleed

Other time

1. Do you suffer with any of the following symptoms related to your menstrual cycle?

Bloating

Low back pain

Pelvic pain

Sleep disturbance

Restlessness

Headaches

Weight gain

Nausea

Anxiety

Clumsiness

Swelling of ankles

1. Are you using any contraception?

Yes

No

1. What form of contraception are you using?
2. Do you take an oral contraceptive pill?

Yes

No

1. Do you have a Mirena or Coil device?

Yes

No

1. Have you ever used Norethisterone to delay a period?

Yes

No

1. Did you have any side effects when taking the Norethisterone?

Yes

No

1. Do you need further advice from any of the following?

Contraception

Regulation of periods during competition

Premenstrual syndrome

Other Female Medical issues
